# Machine Learning in Communication Systems and Networks

**DOI:** 10.3390/s24061925

**Published:** 2024-03-17

**Authors:** Yichuang Sun, Haeyoung Lee, Oluyomi Simpson

**Affiliations:** School of Physics, Engineering and Computer Science, University of Hertfordshire, Hatfield AL10 9AB, UK; y.sun@herts.ac.uk (Y.S.); o.simpson@herts.ac.uk (O.S.)

## 1. Introduction

The landscape of communication environments is undergoing a revolutionary transformation, driven by the relentless evolution of technology and the growing demands of an interconnected world. The proliferation of mobile devices, the rise of IoT applications, and the deployment of 5G networks have ushered in an era where communication environments are not only increasingly complex but also highly dynamic. With the capability of 5G networks to support various forms of vertical integration, the landscape is poised for diverse applications and enhanced connectivity across industries [1]. Furthermore, even for 6G networks, the provision of ubiquitous and 3D coverage in the form of an integrated space–air–ground–sea network is envisioned [2]. In this rapidly evolving technological ecosystem, the need for intelligent solutions to adaptively manage the intricacies of communication systems is more pressing than ever [3]. As we stand on the cusp of these transformative changes, the integration of machine learning techniques emerges as a pivotal catalyst poised to revolutionize the way we address challenges and harness opportunities in communication systems and networks [4].

Traditionally, communication systems heavily relied on model-based approaches, wherein various components were meticulously modeled based on data analysis or measurement data. While these model-based approaches have been successful, they face challenges in accurately modeling dynamic and complex communication environments [5]. Machine learning (ML), capable of extracting characteristics and identifying hidden relationships, becomes a powerful tool in scenarios where traditional designs may falter due to model mismatches [6]. Moreover, the data-driven essence of ML enables inference about network traffic, service requirements, user behavior, and dynamic channels, leading to improved resource provisioning and network operation [3]. ML, with its real-time adaptability and ability to extract insights from vast datasets, promises to reshape communication. The increasing volume and diversity of data in dynamic communication systems demand innovative approaches for efficient operation and optimal performance. From predicting environmental or system status changes to optimizing resource allocation and addressing security threats [7], ML spans applications like intelligent traffic management [8] and automatic reconfiguration in communication infrastructure [9,10].

In this editorial, we explore the intersection of ML and communication, unraveling how these technologies synergize to meet current challenges and leverage opportunities in our highly connected world. In the subsequent section, we provide concise summaries of key points covered in the twenty articles collected in this Special Issue.

## 2. An Overview of Published Articles

In the dynamic realm of communication systems, achieving precise prediction and estimation of communication channels is paramount for optimizing overall system performance. The following five articles concentrate on leveraging ML techniques to effectively address the challenges of channel estimation. In the research conducted by Gaballa et al. (Contribution 1), the primary focus lies in predicting channel coefficients for users in the Non-Orthogonal Multiple Access (NOMA) system. Within the NOMA system, these coefficients assume a critical role in optimizing power distribution at the base station (BS) and streamlining the retrieval of desired data at the user end. The authors employ a deep Q-network (DQN) approach for the BS, enabling it to learn an optimal channel prediction policy. This policy is designed to maximize the sum rates for all users in the NOMA network, leveraging pertinent information such as user states, user distance, channel path loss, and power distribution. Similarly, the study by Gaballa et al. (Contribution 2) delves into channel estimation in power domain NOMA systems. In this investigation, the prediction of channel status information (CSI) is coupled with the determination of power factors for each user, achieved through a Q-learning-based reinforcement learning (RL) approach. In the study by Camana et al. (Contribution 3), the dynamic update of a radio environment map (REM) is explored through the prediction of received signal strength indicator (RSSI) values. The REM proves invaluable in detecting shadow areas with potential for improved network planning and accurate indoor localization. In the study, devices exhibiting similar signal strengths are grouped into clusters using the K-means algorithm, and the dynamic REM update is then orchestrated through a random forest (RF)-based ML algorithm. This model predicts RSSI values for each location, incorporating historical measurement data, including user location and RSSI values. The ML model is designed for real-time updates, facilitated by data collected from a mobile robot, ensuring a seamless and continuous adaptation of the REM, effectively responding to alternations in the wireless environment. The study by Phaiboon et al. (Contribution 4) focuses on path loss prediction within smart agriculture sensor networks, aiming to provide effective coverage areas and system capacity. For challenging environments like plantations, where signal paths are obstructed by trees and vegetation, the authors introduce an adaptive neuro-fuzzy inference system (ANFIS) that combines fuzzy logic and neural networks to learn path loss. Utilizing path loss measurement data and incorporating information such as sensor node distances and antenna heights, the ANFIS model provides an efficient means of estimating path loss. In the article by Ribouh et al. (Contribution 5), the focus is on identifying the distinctive characteristics of the CSI of received signals in vehicular communication by employing convolutional neural network (CNN)-based learning. The study aims to develop a model capable of discerning a vehicles’ surroundings among five categories: rural line-of-sight (LoS), urban LoS, urban nLoS (non-LoS), highway LoS, and highway nLoS. The ultimate goal of this environment detection model is to empower autonomous vehicles to make informed speed limit decisions based on their surroundings.

ML is expected to play an important role in the demodulation process of communication systems since it can adaptively learn and extract complex patterns from received signals, particularly in dynamic and challenging environments. The following four articles are dedicated to the integration of ML into the demodulation process. In the investigation by Harper et al. (Contribution 6), automatic modulation classification (AMC) is used to estimate the modulation scheme employed by the transmitter. AMC proves invaluable in predicting the module schemes of a transmit signal when they are unknown. The authors examine the impact of a variety of architecture changes and propose the design of neural network (NN)-based AMC models. The scenario considered in the study by Zhang et al. (Contribution 7) involves decoding low-density parity check (LDPC) codes. LDPC codes, prevalent in modern communication systems on account of their extended code lengths and versatile combinations, present challenges in decoding and coding blind recognition. To address these challenges, the authors propose an architecture for coding the blind recognition of LDPC codes using deep learning (DL), incorporating a cascade network structure with denoising and blind recognition networks. This innovative approach enhances encoding performance even under poor signal-to-noise ratio (SNR) conditions. In Lamilla et al.’s study (Contribution 8), the attention shifts to a coherent optical encoding system. The authors introduce a robust coding algorithm based on laser intensity profile recognition, utilizing support vector machine (SVM)-based ML for data symbol classification and recognition. This strategy proves effective in mitigating the signal noise added to communication channels. While the above three articles focus on the decoding accuracy performance, the paper by Cho et al. (Contribution 9) considers how to improve the decoding speed for short-length Reed–Muller (RM) codes. Acknowledging the simplistic structure of RM codes and their potential use as control channels in wireless communication, the authors employ a revised auto-encoder scheme, a supervised ML technique, to design an ML-based decoding scheme for faster decoding.

Intelligent resource allocation, empowered by ML, is capable of taking on complex challenges related to the efficiency and adaptability of communication systems and networks. The integration of ML not only ensures the effective utilization of individual resource domains but also facilitates the joint optimization of multiple resource allocations, elevating decision-making processes and overall system performance. The following articles explore ML applications for intelligent resource allocation. The investigation by Pu et al. (Contribution 10) focuses on optimal transmission channel selection in jamming environments. Employing wideband spectrum sensing and Q-learning, the authors design transmitters to dynamically adapt to jamming issues by learning effective channel selection strategies, resulting in high success rates. In Ding et al.’s study (Contribution 11), they employ ML in the routing optimization of low-Earth-orbit (LEO) constellation networks. Satellite nodes, functioning as learning agents, dynamically adapt to changes in topology and channel conditions. Through a collaborative multi-agent reinforcement learning (MARL) framework, satellites share their learning experiences using Q-tables. The proposed three-step routing approach involves neighbor node discovery, followed by offline and online training to ensure that satellites swiftly acquire network link status and adjust their routing strategies accordingly. In the article by Zhang et al. (Contribution 12), the authors delve into the joint optimization of bandwidth and power allocation using multi-agent learning. The proposed approach targets to maximize the system throughput by addressing co-channel interference and ensuring adherence to quality of service (QoS) constraints. Within a large-scale uplink system, individual users act as learning agents, each striving for an optimal strategy in bandwidth and power allocation for their uplink transmission. The collaborative learning process involves sharing users’ past training experiences, leading to the centralized training of all agents aligned with a common objective, maximizing the system’s throughput. In Liu et al.’s work (Contribution 13), aerial edge computing networks, comprising low-altitude aerial base stations (AeBSs) and a high-altitude node, are considered. The study focuses on minimizing task processing delay and energy consumption through the control of AeBSs’ deployment and computation offloading in this two-level aerial network. Utilizing deep RL (DRL), the optimization of low-altitude AeBSs and offloading strategies is carried out by considering factors such as their computational capacity, the number of associated users, the number of computational tasks required by users, and the channel gain with users. Sharing learning model parameters with a high-altitude node, the proposed RL mechanism enables collaborative control among AeBSs, while the high-altitude node serves as a global aggregator, improving training efficiency within the federated DRL framework. In the study by Camana et al. (Contribution 14), a DNN is applied to jointly optimize the beamforming vectors and power-splitting ratios in a multi-input, single-output (MISO) simultaneous wireless information and power transfer (SWIPT) system. The optimization objective is to minimize overall transmission power while ensuring compliance with predefined requirements for energy harvest and minimum data rate within the multi-user system.

ML could also offer benefits for communication network management, including dynamic network configuration, network traffic analysis, and efficient resource allocation. In the article by Hamdan et al. (Contribution 15), Open RAN (O-RAN), recognized for its potential in interoperability, scalability, and cost efficiency, is studied. Despite its advantage, the intricate management of the O-RAN system poses challenges, and the article conducts a thorough survey of current research endeavors while outlining research opportunities about how to uses ML for network automation in O-RAN. In the paper by Baek et al. (Contribution 16), ML is employed to monitor and analyze network traffic, providing benefits in various domains including traffic control, network security, and resource planning. The focus of this paper lies in web services, which are a combination of multiple applications where various application traffic sflows can be intertwined within service traffic. For web services, classifying traffic solely based on service units may lead to high errors in misclassification. To tackle this challenge, a DL-based algorithm performing multitask classification is proposed. This algorithm aims to classify application traffic by considering the relationships between browser, protocol, service, and application tasks within web services.

By leveraging data-driven insights, ML can be useful for service-specific decision making. The following two papers consider distinct service contents, focusing on e-Health and vehicular communication, respectively. In the contribution by AlZailaa et al. (Contribution 17), the emphasis is on addressing the real-time urgency inherent to critical tasks within e-Health applications. Operating within hierarchical fog–cloud networks, the paper employs a support vector machine (SVM)-based ML approach to classify and schedule tasks efficiently. A SVM-based task classification method is introduced, tailored for handling of latency-sensitive critical tasks. Building upon task classification outcomes, the study devises a task priority assignment and resource mapping algorithm. The overarching objective is to minimize latency and enhance the overall resource utilization in fog–cloud networks. In the work by Huang et al. (Contribution 18), the focus shifts to vehicular networks. ML is harnessed for precise vehicle arrival time estimation. Employing support vector regression (SVR)-based learning, the ML model incorporates factors like average vehicle speed, weather conditions, time, and the real-time road traffic information from roadside units (RSUs). Vehicles utilizing this learning algorithm predict their arrival times at specific road sections, transmitting this information to the RSUs. The significance of these data lies in their utilization by RSUs to efficiently manage bandwidth, particularly for supporting reliable real-time video applications. When vehicle users compete for bandwidth, RSUs leverage arrival information to prioritize services, optimizing overall user experiences by offloading traffic to vehicle-to-vehicle (V2V) links.

The traditional approach to analyzing extensive datasets using ML involves centralized ML models. However, the surge in data generation from diverse end devices and concerns over privacy issues have sparked significant interest in federated and distributed learning. Federated learning (FL) allows clients to cooperate to generate a global model without sharing sensitive client data with a server. In the work by Seol et al. (Contribution 19), the impact of statistical heterogeneity indicating non-independent and identical distribution (non-IID) of the training datasets (generated by clients) is highlighted, which clients will use for local training in an FL framework. A novel approach is proposed to reduce statistical heterogeneity and dynamically control batch size and learning rate, aiming to enhance FL performance. In the investigation by Bemani et al. (Contribution 20), the emphasis is on understanding the impact of communication-induced noise during FL training on the convergence and accuracy performance of the ML mode. The paper proposes the use of analog over-the-air aggregation to effectively manage noise in communication channels, ultimately contributing to improved convergence in ML algorithms.

## 3. Conclusions

This compilation of articles sheds light on the transformative impact of machine learning on communication systems and networks. As evident from the diverse range of contributions, ML not only enhances traditional aspects of communication networks but also paves the way for novel applications and optimizations. The showcased articles emphasize the role of ML in addressing intricate challenges, from intelligent resource allocation and dynamic network management to efficient channel estimation and service-specific decision making. The application domains span across e-Health, transportation, agriculture, and more, highlighting the versatility of ML in shaping the future of communication technologies.

Despite significant strides, challenges in applying ML persist. The heterogeneity of communication environments and the ever-evolving nature of network dynamics present ongoing hurdles. Issues related to the privacy, security, and interoperability of ML models in communication contexts also call for further research. Additionally, the scalability and adaptability of ML algorithms to handle the burgeoning volume of data generated in real-time pose continuous challenges.

Looking ahead, collaborative efforts between the ML and communication technology communities will be essential to address these challenges. Interdisciplinary research, harmonization of data formats, standardization of ML methodologies in communication protocols, and the development of scalable, privacy-preserving algorithms will be crucial for the sustainable advancement of ML applications in communication environments.
